# Autophagy in the control and pathogenesis of parasitic infections

**DOI:** 10.1186/s13578-020-00464-6

**Published:** 2020-09-05

**Authors:** George Ghartey-Kwansah, Frank Adu-Nti, Benjamin Aboagye, Amandus Ankobil, Edward Eyipe Essuman, Yeboah Kwaku Opoku, Samuel Abokyi, Emmanuel Kwasi Abu, Johnson Nyarko Boampong

**Affiliations:** 1 Department of Biomedical Sciences, College of Health and Allied Sciences, University of Cape Coast, Cape Coast, Ghana; 2 Department of Medical Laboratory Science, Radford University College, Accra, Ghana; 3 Department of Forensic Sciences, College of Agriculture and Natural Sciences, University of Cape Coast, Cape Coast, Ghana; 4 School of Nursing and Midwifery, College of Health and Allied Sciences, University of Cape Coast, Cape Coast, Ghana; 5 Department of Epidemiology and Biostatistics, State University of New York at Albany, New York, USA; 6 US Food and Drugs Administration CBER, OBRR, DETTD 10903 New Hampshire Avenue, White Oak, USA; 7 Department of Biology Education, Faculty of Science, University of Education, Winneba, Ghana; 8 Department of Optometry and Vision Science, College of Health and Allied Sciences, University of Cape Coast, Cape Coast, Ghana; 9 School of Optometry, The Hong Kong Polytechnic University, Kowloon, Hong Kong

**Keywords:** Autophagy, Autophagosome, Parasitophorous vacuole, Xenophagy, PAAR

## Abstract

**Background:**

Autophagy has a crucial role in the defense against parasites. The interplay existing between host autophagy and parasites has varied outcomes due to the kind of host cell and microorganism. The presence of autophagic compartments disrupt a significant number of pathogens and are further cleared by xenophagy in an autolysosome. Another section of pathogens have the capacity to outwit the autophagic pathway to their own advantage.

**Result:**

To comprehend the interaction between pathogens and the host cells, it is significant to distinguish between starvation-induced autophagy and other autophagic pathways. Subversion of host autophagy by parasites is likely due to differences in cellular pathways from those of ‘classical’ autophagy and that they are controlled by parasites in a peculiar way. In xenophagy clearance at the intracellular level, the pathogens are first ubiquitinated before autophagy receptors acknowledgement, followed by labeling with light chain 3 (LC3) protein. The LC3 in LC3-associated phagocytosis (LAP) is added directly into vacuole membrane and functions regardless of the ULK, an initiation complex. The activation of the ULK complex composed of ATG13, FIP200 and ATG101causes the initiation of host autophagic response. Again, the recognition of PAMPs by conserved PRRs marks the first line of defense against pathogens, involving Toll-like receptors (TLRs). These all important immune-related receptors have been reported recently to regulate autophagy.

**Conclusion:**

In this review, we sum up recent advances in autophagy to acknowledge and understand the interplay between host and parasites, focusing on target proteins for the design of therapeutic drugs. The target host proteins on the initiation of the ULK complex and PRRs-mediated recognition of PAMPs may provide strong potential for the design of therapeutic drugs against parasitic infections.

## Introduction

Autophagy is an important cellular process through which foreign or damaged components that reside in the body are enclosed into organelles for lysosomal decomposition and recycling. Autophagy is conserved evolutionarily in eukaryotes and plays a crucial role in cell metabolism. That is, autophagy offers eukaryotic cells with an opportunity to effectively clear invading pathogens by taking part in immunity [1–3]. Tsukada and Ohsumi’s earlier work on autophagy mutants showed that protein degradation in vacuoles under nutritional deficiency needs autophagy, and at least 15 autophagy genes are involved in the process of autophagy [4]. Autophagy plays important function in disease pathogenesis, like cancer, auto-immune diseases, aging and infection. That is, it aids in cellular clearance of damaged components or pathogens and termed as programmed cell death type II [5–8]. Autophagy (ATG) gene products may be involved in the signal transduction pathway of nutritional starvation. Furthermore, these genes may lead to autophagosomal membrane biogenesis, autophagosomes cytosol isolation, autophagosome transport to vacuoles and subsequent recognition and fusion of autophagosomes and vacuole membrane [4]. However, the dual character of autophagy as in disease inhibition and progression still remains open to discussion [8].

Parasites in the cytoplasm that subvert phagolysosomal degradation usually result in the commencement of autophagy and are cleared via digestion in the autophagolysosome [2, 3]. Accordingly, a number of parasites have acquired skills to impair the autophagic machinery in phagocytes [2]. The protozoan pathogens are evolutionarily divergent parasites including the *Plasmodium* spp. that is responsible for malaria; *Toxoplasma gondii* known for causing toxoplasmosis; *Trypanosoma* spp and *Leishmania* spp. responsible for causing the tritryp diseases [9]. Recent reports show the presence of autophagy during starvation and developments in several pathogens. Starvation-, drug-induced and other stress-related autophagy-like cell death have also been implicated in *Plasmodium falciparum*, *Toxoplasma gondii T. brucei*, *T. cruzi*, and *L. donovani*, indicating a prodeath role of autophagy in these pathogens [10, 11]. Intriguingly, *Leishmania* has acquired the skill to utilize macrophages in order to avoid easy identification and digestion, antigen presentation and further to take advantage of the host cells [2, 12].

For the interplay between pathogens and their host cells to be understood, it is key to also identify the differences existing between starvation-induced autophagy and the other autophagy-related pathways. Thus, it is worthy of note that all kinds of autophagy, including xenophagy, have core machinery and pathway-specific components [13]. The host autophagic system have sequestration tendencies for both parasites subversion of endocytosis and phagocytosis processes. The vesicles in the endolysosomal pathway can fuse with autophagosome containing part or full parasite to provide pathogen ligands for lysosome for clearance or immune activation [14].

The uniqueness of several aspects of the autophagy pathway include the regulatory pathways, autophagosome substrates for sequestration, and possibly the activities and events in autophagosome formation [14]. It is possible for pathogens that usurp host autophagy pathways to have uncommon cellular components with autophagy and are possibly modulated by the parasites in clear distinction. What more, autophagy occurring inside the intracellular pathogen is a new characterized survival strategy for parasites [14]. Thus, autophagy machinery offers a significant approach to parasite growth and development, especially during their liver stages [13]. Although, the Parasitophorous vacuole membrane (PVM) originated from the host cell plasma membrane, the pathogen has extensively modified it by inserting its proteins to the membrane [15]. It is possible that some of these proteins interact with cytosolic defense mechanisms and exploit them. Some of the proteins like Upregulated in infectious sporozoites (UIS)3 and UIS4 are essential during the liver stage [16]. This review indicates relevant studies and current knowledge in autophagy during pathogen infections and further discusses how the uniqueness of autophagy can be utilized for therapeutic purposes.

## Direct role of autophagy in antiplasmodial defense

Macroautophagy, simply referred to as “autophagy” is known to be the most common autophagic process. It depends on the buildup of the double-membrane structure, autophagosome, that engulfs pathogens and further links to lysosomes for degradation [17, 18]. The conservation of autophagy spans from yeast to mammals and is driven by autophagosome. Autophagy is significantly known for providing alternative source of energy in response to starvation, also, it functions as a housekeeper in clearing unwanted proteins and damaged organelles [17, 19]. Furthermore, autophagy clears pathogens by xenophagy [20, 21]. Also, the pathways of autophagy and endocytosis intersect as endosomes interact with autophagosomes to form amphisomes, the intermediate organelles, before linkage with lysosomes [22]. Autophagy occurs mainly during differentiation processes of the life-cycle of Protists. Autophagy is implicated as a mechanism to respond to the stress imposed by the toxic pressure before cellular death [23–26]. Furthermore, there is the likelihood of pathogens manipulating host-cell autophagy for initiation or maintenance of infection within a host [27].

De Brito and colleagues in 1969 found autophagy in *Plasmodium*- infected hepatocytes of humans [28]. They identified malaria pigment as the varied sizes of vacuoles, bound by either one or two membranes [28, 29]. The ATGs are reduced in *Plasmodium* and *Toxoplasma*, the two main protozoan parasites, despite the evolutionary conservation of the machinery for autophagy in most of the eukaryotic organisms. The ATG genes regulate the autophagic process, by triggering the formation of a double-membrane phagophore responsible for engulfing targeted pathogens and organelles [17]. This is coordinated by the Target Of Rapamycin (TOR) complex, acting as an inhibitor [17, 30], and the class III phosphatidylinositol 3-kinase (PI3K) complex, playing a role as a regulator [17, 31]. Since Apicomplexan parasites lack lysosomes, the parasite load in vacuoles is degraded with a proteolytic function [32]. As indicated earlier, the invasive stages of *Plasmodium* parasites in the liver and red blood cells are sporozoites and merozoites respectively, with both lacking food vacuole. Surprisingly, *P. berghei* ATG8 decorated micronemes are removed from the parasite and abandoned by enzymes in the parasitophorous vacuole (PV) [33, 34].

One key protein in the ubiquitin-like regulator of autophagic process termed microtubule-associated protein 1LC3 [35] is associated with the autophagosomal membrane, tightly controlled by the main ubiquitin-like conjugation machinery that is made up of selected ATG proteins, and 16L1 [17]. In autophagy, LC3 -interacting region (LIR) connects with members of the LC3/gamma-aminobutyric acid receptor-associated protein (GABARAP) protein family [13, 36]. That is, the C-terminus of LC3-I is eliminated by the ATG4 protease for a glycine residue exposure. The glycine residue is further linked covalently to the phosphatidylethanolamine, despite the E1, E2, and E3 enzymatic roles of ATG7, ATG3, and ATG12/ATG5-ATG16L1, respectively. Thus, leading to formation of LC3-II [17]. Again, receptor oligomerization increases the interaction with downstream effectors. However, the various autophagy receptors are involved in distinct selective autophagy processes [13, 37]. Several receptors involved in host autophagy act in connection to improve identification and clearance of parasites, leading to immune response [13]. For further functional details of the ATG proteins see Table 1.

**Table 1:** Functions of autophagy-related proteins in parasitic infection

ATG Proteins	Function	References
Atg1 (ULK1) complex (FIP200/Atg101)	Activates the PI3K class III complex	[41–43]
Atg3	E2-conjugating enzyme; autocatalyzes itself to form ATG12-ATG3 complex for maintaining mitochondrial homeostasis	[8, 41]
Atg4	Recycling improperly conjugated Atg8	[41, 44]
Atg7	E1-activating enzyme and protein transport	[41, 45]
Atg8 (LC3)	Membrane elongation and autophagosome closure require covalent attachment of the C-terminal glycine to PE in the phagophore membrane	[41, 46]
Atg9	Golgi-derived membranes supply during initiation to the PAS	[47, 48]
Atg10	E2-like enzyme in ATG12 conjugation with Atg5	[8, 49]
Atg16	Autophagic vacuole formation; Protein transport and degradation	[45, 50, 51]
PI3K class III complex (Vps34, Atg14, Vps15/p150, and Atg6/Beclin1)	Converts PI into PI(3)P at the site of the PAS	[41, 52]
Atg12-Atg5- Atg16	Increase conjugation of Atg5 (Atg 8 in yeast) and autophagosome formation	[52–54]
Atg2-WIPI complex (Atg2-Atg18 in yeast)	Recruitment to the PAS and WD40 repeats for beta propeller formation. Atg18 also prevent Atg4 cleavage	[8, 55]

*PAS* pre-autophagosomal Structure, *PE* phosphatidylethanolamine, *PI3K* Phosphatidyl-Inositol 3-phosphate Kinase, *PI* phosphatidylinositol, *PI(3)P* phosphatidylinositol triphosphate

The parasites cleared by xenophagy are ubiquitinated first, followed by their identification by autophagy receptors and then followed by LC3 protein labeling [13]. The xenophagy’s alternative pathway is LAP, supported by the autophagy machinery. Indeed, LAP has emerged as a useful mechanism in preventing the growth of parasites inside the vacuole [13]. During the process of LAP, LC3 is included directly into the vacuole membrane and function differently from that of the initiation complex Unc51-like kinase (ULK) [38, 39].

Unique autophagy pathways are initiated during *Plasmodium* parasites development in the hepatocytes. Whereas canonical autophagy functions as a unique source of nutrient for *Plasmodium* development in the hepatocytes, *Plasmodium*-associated autophagy-related response (PAAR) is the molecular mechanism relating to both selective autophagy and LAP and further represent an intracellular immune response to the pathogen [13]. Therefore, a number of parasites can be cleared using autolysosome by host autophagy, whereas other group of parasites can usurp the autophagic pathway to its advantage. Therefore, autophagy plays a dual role, both serving as a defense mechanism for the hosts and parasites evasion machinery inside the host cell [40].

## Autophagy compliment the antiprotozoal immune response

Autophagy and immune response are two defense mechanisms for host to resist pathogenic infection [49]. The host mounts a strong and unlimited immune responses with evolutionary pressure on parasites. Many parasites have evolved due to this pressure to prevent direct contact with the host’s immune responses [13]. Interestingly, host cells infected with parasites are well positioned to counter them. Different mechanisms are used in varied approaches against these invaders. Since these response mechanisms are invariably unique and specific to different parasites, intracellular immune response is more appropriate. Significantly, this defense machinery can continue without special linkage with specialized immune cells but are connected via the major histocompatibility complex (MHC) display of peptides obtained from parasites to the adaptive immune system [13].

**Fig. 1:**
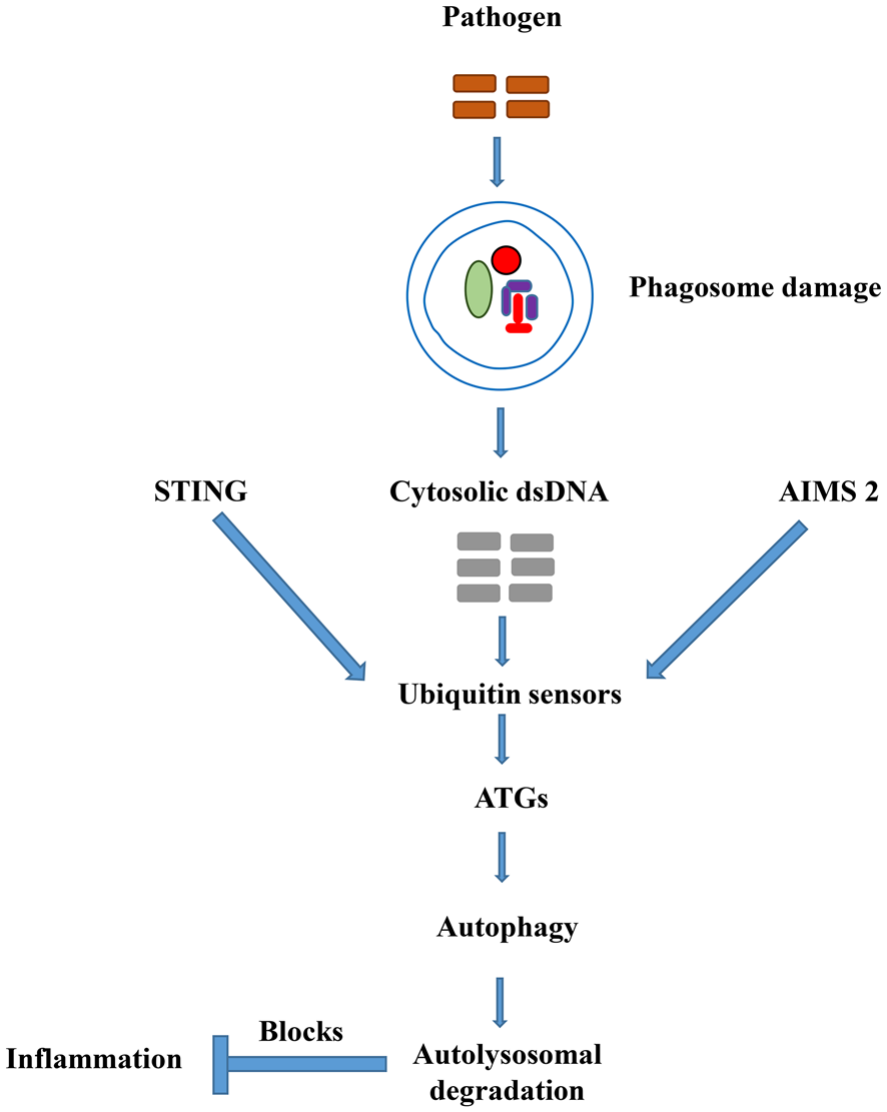
Cytosolic dsDNA induces STING-dependent and AIM2-dependent innate immune responses. STING regulates dsDNA-induced expression of type I IFNs and eliminates invading pathogen. Autophagy is involved in the STING-dependent antimicrobial response. STING-dependent IFN response is negatively regulated by Atg9a. Upon dsDNA stimulation, autophagy is induced in an AIM2-dependent manner and leads to degradation of activated AIM2-inflammasomes, which blocks the immune response

An important role signaling pathways that support survival play is to respond in autophagic manner, which can further be activated regardless of an interferon-gamma (IFN-γ) response (Fig. 1). Stimulator of interferon genes (STING) controls dsDNA induced IFN type I expression and eliminates invasive pathogens. Autophagy actively participates in STING dependent antimicrobial response [56]. Parasite elimination that is dependent on IFN-γ is controlled by the host autophagy machinery independent of the formation of autophagosome [17]. Excepting the *Plasmodium* parasite actin cage polymerization [57], autophagy and its related mechanisms have recently been reported for sporozoites-liver infection as a crucial defense strategy for the host [13]. Like host autophagy, the immune system also plays critical role in cellular and host homeostasis maintenance during pathogenic infection. These, however, show critical cross-talk between these systems [1, 12]. There are distinct and obvious outcomes from the type of parasite and host cells emanating from the host autophagy and pathogens interaction. Many pathogens are overwhelmed and cleared by xenophagy in an autolysosome via autophagic compartments. Other sets of parasites have the capacity to overcome the host autophagy to their advantage. Some of these parasites further prevent the increase in autophagic flux, resulting in non-fusion with lysosomes [27]. There are yet others which have evolved purposely to live and multiply in an autolysosomal characteristics-laced compartment [27]. For instance, distinct effects of two autophagy-related protein ATG10s on microbial-subgenomic replication have been reported. ATG10, a canonical long isoform in autophagy process, can regulate microbial-subgenomic replicon amplification by enhancing autophagosome formation and by combining with and detaining autophagosomes in the periphery of the cellular compartment, leading to limited autophagy flux [49]. Intriguingly, innate immune responses are regulated by two distinct ATG10 versions. That is, both ATG10 and ATG10S can incorporate into autophagosomes and differentially impact autophagosome subcellular localization and autolysosome formation [49].

Autophagy plays a role in different parts of innate and adaptive immunity, among which include, activation of immune system, cells survival, immune cell homeostasis, pathogens clearance or degradation etc. [14]. Autophagy cause the selection and display of peculiar antigens on class II MHC molecules for onward activation of the adaptive immunity [14, 58]. Evidence shows that parasite antigens are engulfed through phagocytosis and further polished in class II MHC compartment (MIIC) which is acidic for presentation to CD4+ T cells [1]. Signaling pathways and innate sensing control autophagy induction, and autophagosomes may provide platform for immune activation [14].

Intriguingly, the clearing of tachyzoites may be as a result of IFN-γ-independent or CD40-dependent autophagy pathway [59]. A frontline defense mechanism against invading pathogens is the pattern recognition receptors (PRRs) identification of pathogen-associated molecular patterns (PAMPs), not forgetting Toll-like receptors (TLRs), and these all important immune receptors have been shown to control autophagy [14]. CD40-induced autophagy is known to be regulated by TRIF and MyD88 signal via TRAF6 in response to macrophage infection by *T. gondii*, in concert with TNF-α [17, 60]. Therefore, it will be intriguing to know if TRAF6 complex plays a role in autophagy-immune activation by uniquely distinct receptors. Further studies are required to give a detailed information regarding PAMPs and PRRs role in regulating autophagy [14], and how TRIF and MyD88 respond to the autophagy machinery and the immune cascade. A study by Choi et al. (2014) shows that Atg5, Atg7, and Atg16L1 are required to control *T. gondii* infection. Furthermore, Atg12-Atg5-Atg16L1 complex is needed for IFN-γ to control *T. gondii* infection [61]. Thus, it is observed that ubiquitin-like conjugation machinery of the autophagy pathway plays significant part in the antiparasitic activity of IFN-γ in that it is needed for the proper targeting of the IFN-γ effectors to the parasites vacuole membrane [61].

Increased quantities of pathogen infection in peritoneal cells in mice lacking autophagy machinery compared to cells from control animals have been recorded, indicating higher infection rate and parasite burden in models without autophagy machinery [27]. There is further suspicion that ATG5 is likely to control the CD4 + T cell cytokine response in dendritic cells via processes different from both canonical autophagy and phagocytosis linked with LC3 machinery or response dependent on the Immune Related GTPase (IRG) [62]. Thus, it can be inferred that autophagic enhancement systematically trigger the infiltration of both neutrophil and helper CD4 T cells via chemokine regulation [58]. These results brings to light the importance of autophagy as a partner to immune responses against some parasites, including *T. cruzi*.

In xenophagy, there is the sequestration of large proportion of the affected cell in autophagosomes that are designated for degradation by lysosome, leading to protection against infection by preventing the survival pathogens [14]. The PVM is a natural fence separating the parasite from the cytoplasm of the liver cell. Although the PVM is a natural fence, the host cells have evolved to identify and clear pathogens within the vacuole. Albeit the pathways for endolysosome and autophagy are essential strategies that control cell balance, host cells utilize these strengths to regulate and clear pathogens. Intriguingly, this digestive capacity strongly affects parasites development inside the liver [13]. Furthermore, as a recycling pathway to source for nutrients during starvation, it is also plausible that pathogens are targeted for clearance by autophagy and further recover molecules that have been attacked by pathogens without destruction to their own essential components and thereby promoting the survival of host cell [14].

## Autophagy and Toll-like receptor signaling in parasitic infections

TLRs are significant parts of innate immunity taking part in the recognition of a different microbial elements. That is, TLRs can be used by macrophages and dendritic cells to discriminate between pathogens and self [63]. Recent reports have shown that a variety of PAMPS, including lipopolysaccharide (LPS) and single stranded RNA (ssRNA), can induce autophagy through different TLRs in immune cells [63–66]. TRIF–p38 is responsible for regulating this pathway but not MyD88 [67]. Therefore, TLR plays an important role in autophagy leading to pathogen clearance. Therefore, it is not surprising that TLR families that combine with the molecular structure of conserved microbial and initiate downstream signal transduction pathways are considered to be the most studied and best characterized PRR family [67, 68]. So far, 10 TLRs have been reported in humans and 13 in mice, respectively [63]. An indication of their involvement in different signaling pathways in mammals. Ligand specificity and subcellular localization are their distinguishing feature. TLR1, -2, -4, -5, and -6 are located at the cell surface, whereas TLR3, -7, -8, -9, -11, and -12 are located in endosomal membranes [69]. Xu et al. showed evidence of close association between TLR-mediated innate immunity and autophagy. Ligands of TLR3 and TLR7 together with LPS activate autophagy. In macrophages, two different ligands of TLR7, ssRNA and imiquimod, regulate autophagosome formation, resulting in LC3 puncta formation [66, 67]. Furthermore, TLRs have been shown to be essential for resistance to protozoan parasites and for detection of components such as *L. major* and *Plasmodium* [70–72]. Specifically, TLR3, - 7 and - 9 (*TLR3/7/9* -*/*-) triple deficient mutant mice were highly sensitive to L. major infection [72]. This shows that the presence of TLR3, -7, and -9 are required for resistance to protozoan parasites. Although the role of TLR2 and TLR4 in malaria remain obscure [73, 74], TLR9 deficient mice were reported to be resistant to PbA-induced cerebral malaria, indicating a role in pathology of TLR9 rather than cerebral malaria protection [75].

Additionally, several examples of potentially important polymorphisms in innate immune genes that affect the outcome of malaria have been identified. These include, but are not limited to TLR4 single nucleotide polymorphisms (SNPs) Asp299Gly and Thr399Ile [76, 77]. These TLR4 SNPs, as well as a Thr1486Cys polymorphism in TLR9, have been linked to increase risk of low birth weight and maternal anemia [78]. In spite of the obscure nature of the mechanisms for TLR4 polymorphisms, the 399 SNP is suggested to be predispose to severe malaria. This indicates that TLR-4 helps in parasite recognition and host responses [76]. Further report indicates that two TLR9 SNPs (TLR9 C allele at -1237 and G allele at 1174) are associated with increase in IFNγ levels in children with cerebral malaria [79]. Intriguingly, Khor and colleagues have reported on a common SNP in the TLR adapter known as TIRAP (TIR domain containing adaptor protein) or Mal (MYD88 adaptor-like) [63]. As expected, treatment with E6446, a synthetic antagonist of nucleic-acid-sensing TLRs, was reported to minimize the activation of TLR9 and stopped the exacerbated cytokine response during cerebral malaria [63]. Thus, supporting the hypothesis that nucleic acid sensing TLRs are required in the development of ECM [31].

Also, macrophages deficient in the TLR2 and NOD/receptor-interacting protein 2 (RIP2) pathways show defective autophagy induction and fail to co-localize bacteria within autophagosomes in response to *Listeria monocytogenes*. A different report showed that induction of phagocytosis and autophagy is as a result of *Staphylococcus aureus*-mediated stimulation of TLR2 in mouse macrophages [80]. In summary, these data suggest that the presence of microorganisms in vivo can stimulate TLR2 and mediate autophagy induction to promote pathogen clearance [67].

## Parasite evasion and modulation of host autophagy

Autophagy plays a number of significant roles, including amino acid pool maintenance during nutrient limitation, anti-ageing prevention, suppression of tumours, neurodegeneration and immunity regulation [22]. Autophagy also serves as defense mechanism against pathogens, and as a result, several microbes are cleared through this pathway. Despite microbe elimination role by autophagy, several parasites use different mechanisms to overcome host autophagy and establish replicative niches [22, 81]. These steps can be classified into three procedures: prevention of autophagy induction by inhibiting host ATG proteins; downstream autophagy degradation pathway interference; and subversion of host autophagy to support pathogen replication [1, 2].

It is established that PV within macrophages harbors *T. gondii* and blocks the parasites-lysosome fusion [14]. The approaches used by the pathogens to subvert the immune responses and the resultant interplay leading to autophagy from degradation of the vacuole are significant to the outcome of infection [82]. As the parasite lives within a PV during its development in the liver, the immediate membrane surrounding it present as the main barrier to the cytoplasm of the liver cell [22, 57]. The PVM is modified into a continuous, membrane-bound system called tubovesicular network (TVN) formed into the host cell’s cytoplasm [13]. Intriguingly, this PVM contribute significantly towards the subversion of the host autophagy machinery by the parasites [57]. Therefore, the PVM becomes obsolete and disintegrates after the asexual replication giving rise to several merozoites [13, 83].

Surprisingly, recent report indicates that mutant parasites with functional deficiency in PVM are still capable of infecting the hepatocytes, but insignificant amount further develop into the next stage. Also, electronic microscopic study has indicated genetic excision of the two 6-Cys sporozoite proteins P52 and P36 result in free living in the host cytoplasm [84]. Albeit some mutant parasites deficient in p52/p36- are able to complete development in the liver with reduction in survival rate [85, 86]. Therefore, this strongly shows that parasites require PVM for protection against host cell attacks while still offering window of opportunity for nutrients acquisition for parasite growth and development [13]. Parasites inability to subvert immune response is due to a permanent association between LC3 and PVM [87, 88]. Thus, parasites need to stimulate membrane shedding from the PVM towards TVM so as to detach PVM-associated autophagy proteins [13, 88]. Therefore, the role of the prevention of membrane shedding of the parasite needs further interrogation.

Different *Leishmania* species are linked to autophagy induction. This serves as a platform for obtaining critical nutrients for parasite growth and development [16]. Indeed, finding the mechanism employed by *Leishmania* species to subvert the host autophagy machinery [16] will be key to unravelling the mystery. The mammalian TOR complex 1 (mTOR)-independent autophagy induction upon *L. major* infection [2] contradicts a recent report indicating the inactivation of mTOR by protease GP63 derived from parasite [89]. The observed discrepancy is likely due to the distinct experimental setup, Whereas Franco and colleagues used WT BALB/c mice [2], Jaramillo et al.[89] utilized bone marrow-derived macrophages (BMDM) from BALB/c mice deficient in Src homology region 2 domain-containing phosphatase-1 (SHP-1). This and other differences in the experimental setup may have led to the varied results. Also, induction of autophagy offer protection to *L. amazonensis* leading to increased intracellular load similar to lipid bodies formation and production of PGE 2 from BALB/c macrophages [12]. Autophagy stimulation further causes IFN-γ to increase *L. amazonensis* infection levels. Treatment with either wortmannin or 3-MA can be used to avert damages caused by IFN- γ. Also, transmission electron microscopy has shown that IFN- γ treatment of infected macrophages led to the formation of vesicles with double-membrane and autophagosomes-like structure [12]. Intriguingly, BALB/c macrophages treatment with IFN-γ after recovery from autophagy resulted in parasite clearance. This indicates the possibility of diverse roles of IFN-γ on infection, depending on the prevailing conditions of the signaling pathway of host cell. Interestingly, parasite including *L. major* and *T. cruzi* in the macrophages of BALB/c were not affected by autophagy induction. Therefore, the role of autophagy as an effective cellular mechanism against a specific or confined set of parasites cannot be underestimated [12].

*Plasmodium* liver stage is offered additional nutrient by host cell’s canonical autophagy. Further interference of the host macroautophagy pathway indicates an overwhelming parasite growth reduction [22, 87, 90]. Cells deficient in ATG5- have defect in initiation of, xenophagy or LAP, because the ATG5 protein is involved the LC3-lipidation pathway [13]. In spite of the deprivation of nutrient of parasites in host cells deficient in ATG5 and thus lead to a retarded growth and development, their gross survival rate is remarkably improved since the PAAR response also depends on ATG5 protein [87, 90]. However, host cells deficit for focal adhesion kinase family interacting protein of 200 kD (FIP200) misses the canonical autophagy pathway. As a result of reduction in nutrient supply, parasite growth is highly affected. Since FIP200-deficient cells still have responses from PAAR, parasite survival rates are similar to their wild-type counterpart [13, 90]. In sum, the liver stage *P. berghei* parasites appear to be supplied with nutrient from canonical autophagy but are target of PAAR responses [13]. The complexity of autophagy machinery and pathway makes drug design difficult, however, targeting both ATGs and PAAR responses will be key to unlocking the code for possible elimination of these dreaded parasites.

## Roles and mechanisms of autophagy as a therapeutic consideration

Parasites have acquired several adaptive responses to environmental changes including immune response from the host. The survival of parasites is dependent on the parasite-host interaction cycle [27], leading to common human disorders classified as parasitic diseases. Several of these disorders are grouped as Neglected Tropical Diseases (NTDs) due to the presence of the causative agents mainly in tropical and sub-tropical areas, and as such are of less importance to the drug manufacturing companies and even the relevant international health institutions [27]. It has been observed that the acidic pH of lysosomes is crucial for their function. In this context, several reagents used to estimate lysosomal degradation, such as ammonium ion, chloroquine and bafilomycin, can also prevent autophagy protein degradation. However, these reagents may affect a series of other cell functions, limiting their use in this respect [91]. To develop new therapeutic agents, it is important to understand the host-parasites interaction at various stages including uptake, differentiation, replication, and release [2].

Autophagy has emerged as potential drug target for a several disorders or diseases, including parasitic diseases [14]. Activation of autophagy by the use of pharmacological or immunological processes can enhance autophagy of pathogens. However, the complexity of the interaction between autophagy and various parasites raises a number issues in line with therapeutic remedies for infectious diseases [13, 14]. Thus, protozoan infections present a more complex situation, where host and pathogen autophagy may have mutual benefits, such that therapy in these cases may require specific targeting for different species [14]. Two key opposing regulators of autophagy acknowledge the metabolic state of the host cell: the AMP-activated protein kinase (AMPK) and the mTORC1, serving as activator and inhibitor respectively. These two regulators act as molecular switches through phosphorylation-dependent manner to control the initiation complex ULK [13, 92]. The activation of the ULK complex causes the initiation of autophagy response [13]. Chemical and immunological induction lead to some parasites susceptibility to autophagy. Rapamycin inhibits mTOR and activates autophagy, respectively and improves *T. gondii* targeting for autophagolysosomal degradation [14]. In developing new anti-leishmanial drugs, it is imperative to consider the role host macrophages play in *L. major* parasites survival from digestion by host autophagy, and also identify the putative regulatory machinery of autophagy [2]. The PV-LC3 protein enhancement for *T. cruzi* and the presence of inhibitors of autophagy such as wortmannin, 3-methyladenine and vinblastine slows the recruitment and reduction of parasitic infection. Interestingly, lack of some genes specific to autophagy which are needed for autophagy initiation causes a reduction in infection, implying that compartments derived via autophagy are needed for parasite entry into the host cell [27]. Therefore, using certain unique strategies to target parasite antigens to autophagosomes may improve the efficacy of vaccines, just as antigen-membrane protein complex of autophagosome in the case of influenza virus, LC3, offer synergistic improvement of CD4+ T cell responses than in the case of only antigen [14].

In eliminating *Toxoplasma* infection, there is the need for synergistic interaction between CD40 ligation and TNF α, further stimulating a signal involving Beclin1 and ULK1 to enhance *T. gondii* autophagic clearance [16, 93]. Therefore, any drug that can enhance the connection between CD40 and TNF α will be the key to the search for therapeutic drugs for toxoplasmosis and other protozoan infections. A recent study proposed another mechanism in activated cells with no CD40 whereby there is activation of focal adhesion kinase (FAK)-Src—Epidermal growth factor receptor (EGFR) transactivation by *Toxoplasma* invasion to Signal transducer and activator of transcription 3 (STAT3) pathway. This further inhibits formation of autophagosome and thus cause parasites destruction [94], thereby being a potential therapeutic target. In addition to the above reports, a new finding indicated an EGFR inhibitor, Gefitinib, played an important role in the decline of parasite increase in HeLa cells [95]. IFN-γ causes upregulation of Guanylate Binding Proteins (GBPs) and IRGs expression in host respectively, both of which are required for disruption of pathogen vacuoles [16]. A reliance on the IRG Irgm3 localization to the membranes of autophagosome protecting the parasite has been reported. Furthermore, Irga6 and Irgb6 involvement with macrophage PV, granulocytes and fibroblasts needs Atg5 [16]. In host cytoplasm, the absence of Atg5 leads to Irga6, Irgb6 and Irgd. In the PV, there is recruitment of Atg7 and Atg16L1 together with Irgb6 and mGBPs [16]. In addition, regulation of *Toxoplasma* infection and IRGs and mGBP2 loading onto the PVM requires Atg3 [61]. However, there is the need for additional work to address the interplay between autophagy and *Toxoplasma*. Since autophagy is associated with multiple pathways depending on a number of factors including, host, cell type and strain of parasite, an enhanced and integrated approach will provide the needed answers/solutions to the number of raging questions [40]. UIS3 is leading vaccine candidates against malaria, acting as a potent and important regulator of autophagy evasion by *Plasmodium* parasites [96, 97]. The proposition that UIS3 and host LC3 interplay represents a target for antimalarial therapeutic development is, therefore, considered [16]. Recent report indicate that UIS3 can provide protection against chimeric *P. berghei*–*P. falciparum* parasites in multiple strains of mice [97, 98]. It is further believed that UIS3 likely provides protection through antigen-specific CD8+ T cells and that coadministration of UIS3 with the vaccine candidate ME-TRAP results in 100% sterile protection in BALB/c mice [97]. For instance, in *P. vivax*-liver infection, enhancement of LC3 and lysosome role towards the PVM is triggered by stimulation of interferon-gamma (IFN-γ) [96].

Since the argument involves double-edged sword role of the host autophagy; that is for both cell survival or death during infection at the liver stage [99], there is an emerging idea that PAAR could be represented by the development of *Plasmodium* in the liver [13, 16, 90]. Upon *Plasmodium* infection, conditions leading to the conjugation system of LC3 and the lipidated LC3 enlistment to the PVM are still vague [16]. Although, UIS3 protein from the parasite binds to and holds LC3 firmly on the PVM [100]. Upon sequestration, UIS3 prevent LC3 linkage with the rest of targeted proteins, leading to inhibition of autophagy [100]. New insight into the LC3-UIS3 interaction and the importance of UIS3 for parasite survival and development will be helpful in our quest to understand the LC3-UIS3 protein–protein interplay. Also, engagement of LC3 on the PVM through parasite UIS3 protein on autophagy machinery of the host and the response by PAAR could be used for therapeutic advancement [100].

The innate immune signaling regulation may be mediated by the autophagy response from the host, as well as parasites clearance, and the improvement of antigen presentation in adaptive immune response. Thus, triggering the autophagy pathway is of therapeutic interest for parasitic disease elimination [101]. Despite the myriads of preclinical data supporting the autophagy role in parasite elimination, there are currently no specific molecule for humans use [102]. In spite of this challenge, determining the molecular basis of the evasion strategies of parasites and using them for treatment purposes is still possible and urgently needed. Interestingly, there is renewed interest in understanding of autophagy regulation in *Plasmodium* species emanating from the recent work involving artemisinin-resistance mutations in PfAtg18 [103]. In addition, resistance to chloroquine is also associated with changes in PfATG8 distribution [33, 104]. Since knockouts in *Plasmodium* Atg8 are difficult, a selective prevention of Atg8 lipidation would be an essential tool for potential antimalarial therapeutic [41].

Elucidating such functions of autophagy proteins in sensing and inducing the breakdown of pathogenic structural membranes in the cytoplasm might lead to novel therapeutic and/or prophylactic treatments for these pathogens. It will further offer greater insight and understanding of fundamental knowledge in the autophagy pathway [61].

## Conclusion

Not only the evolutionary ability of autophagy to clear intracellular pathogens has been retained but also the fine-tuning to improve and control a number of antiprotozoal immune responses. In spite of our better understanding of autophagy in antiprotozoal immune response, many unanswered questions still need to be addressed. These include proper understanding of the complexity that exists between the immune processes autophagy has been associated with and the parts of the ATG machinery involved. Recently, significant interventions has been made, dissecting the function of the ATG machinery in *Plasmodium* and other protozoan infections, however, several questions remain unanswered. The plethora roles of the PVM is partly due to its complexity in design and creation of compartments that leads to the development of the TVN extension. Significantly, the PVM/TVN interferes with parasite-host cell interactions. A major engagement is to decipher the divergence at the molecular level between the various parasite types that result in unique and specific response by autophagy. Our challenge further will be to identify the molecular mechanism detailing parasite’s invasion and evasion strategies and use them for therapeutic advantage. Autophagic mechanism from the host has been implicated in the regulation of parasite invasion, further affecting the maturation of the PV. Thus, controlling strategies could lead to the regulation of infection by the host cell. In addition, LC3 recruitment to the PV by UIS3 results in an intriguing findings that require a better understanding few years to come.

## Data Availability

All materials have been included.

## Abbreviations

AMPK: AMP-activated protein kinase
ATG: Autophagy-related protein
BMDM: Bone marrow-derived macrophage
EGFR: Epidermal growth factor receptor
FAK: focal adhesion kinase
FIP200: Focal adhesion kinase family interacting protein of 200 kD
GBP: Guanylate Binding Protein
IFN-γ: Interferon-gamma
IRG: Immunity Related GTPase
LAP: LC3-associated phagocytosis
LC3: Light Chain 3
MHC: Major histocompatibility complex
MIIC: MHC class II compartment
mTOR: Mammalian TOR
PAAR: *Plasmodium*-associated autophagy-related
PAMP: Pathogen-associated molecular pattern
PRR: Pattern recognition receptors
PV: Parasitophorous vacuole
PI3K: Phospoinositol 3-kinase
PVM: PV membrane
SHP-1: Src homology region 2 domain-containing phosphatase-1
STAT3: Signal transducer and activator of transcription 3
STING: Stimulator of interferon genes, TLR: Toll-like receptors
TOR: Target of rapamycin
UIS: Upregulated in infectious sporozoites
ULK: Unc51-like kinase
